# Effects of six months of Yoga on inflammatory serum markers prognostic of recurrence risk in breast cancer survivors

**DOI:** 10.1186/s40064-015-0912-z

**Published:** 2015-03-26

**Authors:** Dorothy Long Parma, Daniel C Hughes, Sagar Ghosh, Rong Li, Rose A Treviño-Whitaker, Susan M Ogden, Amelie G Ramirez

**Affiliations:** Department of Epidemiology and Biostatistics, Institute for Health Promotion Research, University of Texas Health Science Center at San Antonio, 7411 John Smith Drive Suite 1000, San Antonio, TX USA; SRA International, Inc., Fairfax, VA USA; Department of Molecular Medicine, Institute of Biotechnology, University of Texas Health Science Center at San Antonio, San Antonio, TX USA

**Keywords:** Inflammation, Yoga, Breast cancer survivors, Exercise, Biomarker

## Abstract

Yoga-based exercise has proven to be beneficial for practitioners, including cancer survivors. This study reports on the effect on inflammatory biological markers for 20 breast cancer survivors who participated in a six-month yoga-based (YE) exercise program. Results are compared to a comprehensive exercise (CE) program group and a comparison (C) exercise group who chose their own exercises.

“Pre” and “post” assessments included measures of anthropometrics, cardiorespiratory capacity, and inflammatory markers interleukin 6 (IL-6), interleukin 8 (IL-8), tumor necrosis factor alpha (TNFα) and C-reactive protein (CRP). Descriptive statistics, effect size (*d)*, and dependent sample ‘t’ tests for all outcome measures were calculated for the YE group.

Significant improvements were seen in decreased % body fat, (−3.00%, *d* = −0.44, p = <.001) but not in cardiorespiratory capacity or in inflammatory serum markers. To compare YE outcomes with the other two groups, a one-way analysis of co-variance (ANCOVA) was used, controlling for age, BMI, cardiorespiratory capacity and serum marker baseline values. We found no differences between groups. Moreover, we did not see significant changes in any inflammatory marker for any group.

Our results support the effectiveness of yoga-based exercise modified for breast cancer survivors for improving body composition. Larger studies are needed to determine if there are significant changes in inflammatory serum markers as a result of specific exercise modalities.

## Introduction

Each year**,** over 226,000 new women are diagnosed with breast cancer (American Cancer Society 2012). Breast cancer remains the most prevalent cancer for women**,** and for Latina women, it is still the number one cause of cancer mortality (American Cancer Society 2009). A growing body of research documents the benefits of exercise for breast cancer survivors, including improvements in fitness, physical functioning, fatigue and emotional well-being (Courneya 2003; Courneya et al. 2003; Segal et al. 2001; Pinto et al. 2005; Schmitz et al. 2010). Indeed, cohort studies have shown a decreased risk of breast cancer recurrence and lowered breast cancer-specific mortality for survivors who are more physically active (Holmes et al. 2005; Irwin et al. 2011; Ballard-Barbash et al. 2012; Patterson et al. 2010). Thus, engaging in exercise activities is an important behavior for breast cancer survivors (Schmitz et al. 2010; Courneya et al. 2000).

Although these benefits have been well documented, only a minority of breast cancer survivors are active at levels consistent with public health guidelines (Schmitz et al. 2010). Like others who experience cancer, many breast cancer survivors who were not active before diagnosis stay inactive; and, those who were active often do not return to their previous level of activity (Schmitz et al. 2010). Specifically, approximately four out of every five breast cancer survivors do not meet national exercise recommendations at 10 years post diagnosis (Mason et al. 2013).

For centuries, yoga has been recognized as a form of exercise that can yield increased flexibility, lipid profile management, strength and endurance for regular practitioners (Olivo 2009; Ulger and Yagli 2011; Pullen et al. 2008; Gordon et al. 2008; Agte et al. 2011; Phoosuwan et al. 2009). Yoga-based exercise is also emerging as an important practice to be used for cancer survivors, and has been shown to improve survivors’ self-reported quality of life (QOL) (Culos-Reed et al. 2006; DiStasio 2008; Banasik et al. 2011; Danhauer et al. 2009; Bower et al. 2012; Buffart et al. 2012; Lengacher et al. 2012; Ulger and Yagli 2010; Van Puymbroeck et al. 2013; Moadel et al. 2007; Mustian et al. 2013; Kiecolt-Glaser et al. 2014).

Though most randomized trials using yoga-based exercise have looked at QOL parameters as primary outcomes (Buffart et al. 2012), one recent study also looked at inflammatory markers interleukin-6 (IL-6), tumor necrosis factor alpha (TNF-α), and interleukin-1 beta (IL-1β), with significant improvement in IL-6 and IL-1β (Kiecolt-Glaser et al. 2014). For this paper, we examined TNFα, IL-6, IL-8, and C-reactive protein (CRP). Previous studies have shown that these biomarkers may be affected by physical activity, and may be involved in biological mechanisms associated with cancer recurrence and prognosis (McTiernan 2008; Allin et al. 2011).

Exercise along with a low-caloric diet can decrease serum levels of certain inflammatory cytokines like TNFα, IL-6 and CRP (Bruun et al. 2006). Some studies suggest that blood levels of TNFα may be reduced with exercise training (You et al. 2004; Straczkowski et al. 2001). Physically active and fit individuals have lower levels of IL-6, CRP and other inflammatory markers (Abramson and Vaccarino 2002). Both IL-6 and IL-8 are regulated by exercise (Frydelund-Larsen et al. 2007). IL-6 and TNFα are produced by adipose tissue, and are elevated in the serum of obese individuals. The loss of body fat through exercise could thus be a mechanism for inflammation reduction (Kern et al. 1995; Mohamed-Ali et al. 1999; Fried et al. 1998). Lynch and colleagues found that diet plus exercise decreased CRP in postmenopausal women (Lynch et al. 2011). A study in prostate cancer survivors found that circulating levels of CRP statistically and clinically decreased in the exercise group and increased in the control group (Galvao et al. 2010).

Allin and colleagues found that higher CRP levels at the time of breast cancer diagnosis are associated with decreased chances of overall and disease-free survival (Allin et al. 2011). IL-6 may also be a marker for breast cancer recurrence (Knupfer and Preiss 2007). Elevated serum levels suggest poor prognosis and poor survival rates in breast cancer patients (Knupfer and Preiss 2007; Hong et al. 2007). Elevated serum IL-6 has been linked to tumor progression and growth (Paduch and Kandefer-Szerszen 2005; Gao et al. 2007). An IL-8 polymorphism has been associated with increased breast cancer risk by a recent meta-analysis (Wang et al. 2014). Conversely, an inverse relationship has been found between IL-8 expression and metastasis and/or local recurrence, highlighting the complex role of this cytokine in breast cancer progression (Zuccari et al. 2012; Todorovic-Rakovic and Milovanovic 2013).

Based on these initial studies that provide strong evidence of the benefits of purposeful yoga-based exercise, and to better understand the effects of different modalities of exercise on a more comprehensive array of inflammatory markers, we sought to conduct a six-month randomized trial comparing yoga-based exercise with “conventional” exercise and with exercise of the individual’s own choosing. We randomized 94 post-treatment breast cancer survivors either to a yoga-based exercise program (YE), a “conventional” comprehensive exercise (CE) program (aerobic, resistance, flexibility) consistent with oncology exercise guidelines (Schmitz et al. 2010; Doyle et al. 2006) or to a comparison group (C) where participants chose their own exercise activities. Here we focus on the outcomes for the YE group and compare the results to the other two groups.

## Methods

### Recruitment

After obtaining approval from the Institutional Review Board at The University of Texas Health Science Center at San Antonio (UTHSCSA), and the Cancer Therapy and Research Center Protocol Review Committee, participants were recruited with assistance from the ThriveWell® Cancer Foundation’s DIVA (Deriving Inspiration and Vitality through Activity) program, a self-referral program that offers support services for breast cancer survivors. Potential participants who called in to register for DIVA services or expressed interest in response to study flyers, radio and TV advertisements were screened for eligibility by research staff. Inclusion criteria were: age 18 or older; previous diagnosis of invasive breast cancer or ductal carcinoma *in-situ*; being at least two months post-treatment (surgery, chemotherapy, radiation, or any combination thereof); able to provide informed consent; and free of any absolute contraindications for exercise testing as stated in the American College of Sports Medicine (ACSM) Guidelines for Exercise Testing (American College of Sports Medicine 2013). If interested in participating in the research, participants were asked over the phone to complete the Physical Activity Readiness Questionnaire (PAR-Q), as detailed in the ACSM’s Guidelines for Exercise Testing and Prescription (American College of Sports Medicine 2013). Physician’s clearance was required for all participants that answered “yes” to any of the seven questions listed on the PAR-Q prior to scheduling them for baseline appointments. Participants who answered “no” to all questions or had received physician’s clearance were scheduled for a baseline assessment. Participants were asked to provide a detailed list of all current medications at baseline assessment; any participants who were on maintenance therapies (e.g. Tamoxifen) were allowed to participate in the study.

### Study design

**Figure 1:**
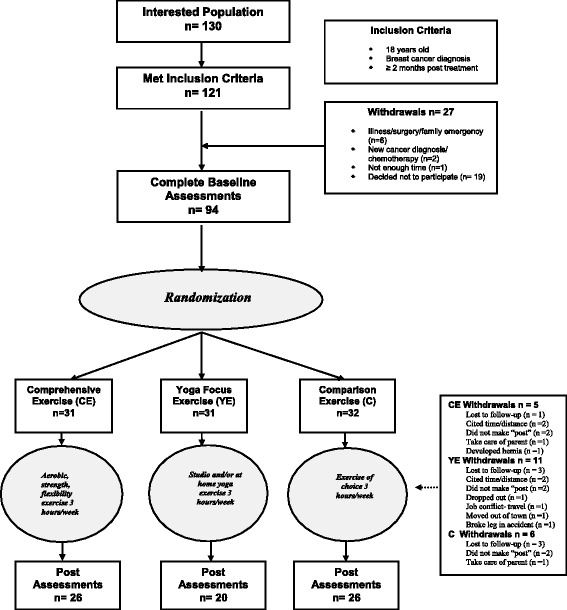
**Study flow diagram.**

Of the 130 women who expressed interest in the study, 121 met the inclusion criteria, and 94 of those completed baseline fitness assessments (Figure 1). Informed consent was obtained with the baseline assessments conducted at a cancer treatment center in the San Antonio, Texas area. Using a minimization adaptive randomization technique, participant covariates of age, body mass index (BMI), and cardiorespiratory capacity (estimated VO_2_max) were used to assign 94 participants either to 1) a yoga-based exercise program (YE) group, n = 31; 2) a comprehensive, individualized exercise program (CE) group, n = 31; or, 3) a comparison group (C), in which participants performed exercises of their choice, n = 32. Of the 31 participants randomized to the YE group, 20 completed the 6-month trial and completed “post” fitness assessments. In the other two groups, a total of 11 participants dropped out, resulting in 26 participants completing the study in the CE group and the C group, respectively. There were no reported injuries in any group related to the exercise programs.

Specifically designed yoga exercise classes were taught to participants in a local yoga studio. YE participants were asked to attend three 1-hour yoga classes per week. Participants were neither encouraged nor discouraged from seeking other forms of exercise but were highly encouraged to attend the yoga classes offered. In addition, an audio CD and an instruction booklet for the specific protocol were provided to the participants for use at home when class attendance was not feasible. For the CE group individualized exercise programs were prescribed by an ACSM certified Clinical Exercise Specialist®. The program components were based on the participants’ individual baseline fitness results, following ACSM guidelines (American College of Sports Medicine 2013), and consistent with the levels of activity as described in the public health guidelines for physical activity for adults (United States Department of Health and Human Services 2008; Haskell et al. 2007) taking into account the participants’ breast cancer survivor status (Schmitz et al. 2010; Doyle et al. 2006). CE programs included components of aerobic, resistance and flexibility training focused on three 1-hour sessions per week. C group participants were asked to participate in three hours of exercise of their own choosing, though they were encouraged to attend DIVA activity classes (each class is approximately one hour). CE and C participants were asked to log their activities. Similar to the YE group, CE and C participants were neither encouraged nor discouraged from seeking other forms of exercise beside those prescribed. An assigned research staff member called all participants every two weeks to answer any questions, monitor possible safety concerns, and encourage program participation.

### Fitness assessments

The fitness assessments included tests for cardiorespiratory capacity and body composition. For cardiorespiratory capacity, a ramped cycle ergometer test based on ACSM Guidelines for submaximal exercise testing (American College of Sports Medicine 2013) was conducted to obtain estimated VO_2_max (mlO_2_/kg/min) based on a linear heart rate (HR) response to increased VO2 uptake. A Lode Corival Cycle (Groningen, Netherlands) and a ParvoMedics TrueOne® 2400 metabolic cart (ParvoMedics, Sandy, UT) were used to obtain gas exchange data.

Anthropometric measures included BMI (kg/m^2^) calculated from height (cm) and weight (kg), and % body fat estimated from three-site skinfold measure. Height (cm) and weight (kg) were measured using a wall-mounted Stadiometer (Seca 644 Handrail Scale). For % body fat estimate, three-site (triceps, supra-ilium, and quadriceps) skinfold assessments were performed according to ACSM guidelines (American College of Sports Medicine 2013). Calipers (Lafayette Instruments, Lafayette IN) measured the skinfold tissue in mm with duplicate measurements taken at each site. Unless contraindicated by lymphedema, recent surgery, or participant preference, all measurements were taken on the right side of the body. Skin fold measurements were summed. Body density (Db) and % body fat were calculated using ACSM-recommended formulas (American College of Sports Medicine 2013):

$$ \begin{array}{l}Db = 1.099421\ \mathit{\hbox{--}}\  0.0009929\ \left( skinfold\  sum\right) + 0.0000023\ {\left( skinfold\  sum\right)}^2\mathit{\hbox{--}}\  0.0001392\ (age).\\ {}\%\  body\  fat = \left( 4.96/Db\right)\mathit{\hbox{-}} 4.51.\end{array} $$

Participants received a $25 gift card as compensation upon completion of each assessment.

### Yoga program specifics

A structured Hatha yoga exercise program was developed specifically for this study. The program took into account potential limitations of limb movement, higher body fatness, and the lower aerobic and strength conditioning characteristic of post-treatment breast cancer survivors (Schmitz et al. 2010). The protocol was developed by an experienced yoga instructor, a licensed clinically trained physical therapist (BS and MS), with experience working with cancer survivors. The protocol and sequencing of postures were designed with a great deal of specificity to guarantee that the subjects would receive the same instructions and perform the same routine, regardless of the instructor or class attended. Modifications were developed for each posture to accommodate the limitations that might be encountered within this population. The instructors that led the classes for the study participants received training in specific language to be used, as well as timing/pacing for the class to ensure consistency for the 60-minute program used throughout the duration of the research study. Participants also received an audio CD and booklet detailing the yoga program with photographs and instructions to be used at home when they were unable to attend class.

### Measures

#### Co-morbidity index

From the medical history information, a co-morbidity index was calculated with a sum score of the number of a possible 17 items endorsed: (diagnosis of a heart attack, heart failure, heart condition, circulation problems, blood clots, hypertension, stroke, lung problems, diabetes, kidney problems, rheumatoid arthritis, osteoarthritis, anemia, thyroid problems, neuropathy, fibromyalgia and hepatitis.)

#### Inflammatory markers

Collected serum was aliquoted into 400-μL cryovials labeled with participant Study ID and stored in a −80°C freezer. All samples were analyzed in batch at the Core for Advanced Translational Technologies (CATT) laboratory in the Dept. of Molecular Medicine/Institute of Biotechnology at UTHSCSA. The Luminex (Austin, TX) FlexMap 3D (FM3D) platform was used to analyze participant sera for inflammatory markers of interleukin 6 (IL-6), interleukin 8 (IL-8), tumor necrosis factor alpha (TNFα) and C-reactive protein (CRP). Milliplex kits specific to each analyte -- Human Cytokine Panel 1 (IL-6, IL-8, TNFα) and Human Neurodegenerative Panel 2 (CRP) -- were purchased from Millipore (Billerica, MA) to perform the assays. Serum samples were thawed and 25ul of each sample run in duplicate on a 96-well plate with blanks, standards, and assay controls. Depending on the targets for each kit, samples were diluted per manufacturer’s protocol. Detection limits were as follows: IL-6, 0.96 - 15,000 pg/mL; IL-8 and TNFα, 0.64 - 10,000 pg/mL; CRP, 0.012 – 50.0 ng/mL. Raw results from the Luminex FM3D for serum levels of IL-6, IL-8, TNFα and CRP falling below detection limits were deleted.

### Treatment of data

All analyses were performed using SPSS. Descriptive statistics were performed on all variables (range, mean, standard deviation). Paired-sample “*t*-tests” were performed to compare “pre” and “post” values. Because we were also interested in the magnitude of change for the YE participants, in addition to statistical significance, we calculated effect size as ES = (m1-m2)/s1 where m1 = “pre” mean, m2 = “post” mean and s1 = “pre” standard deviation. Effect sizes were defined as small (0.2), medium (0.5), or large (0.8) (Cohen 1988). To compare YE participant outcomes with the other two groups, we used a one-way analysis of co-variance (ANCOVA), controlling for co-variates of BMI, cardiorespiratory capacity and baseline value for each serum marker. Bonferroni *post hoc* tests were applied when the difference was significant (*p <* 0.05) according to the results of the ANCOVA. Descriptive analyses were conducted on the original units of the serum marker and values were log-transformed (LN) by taking the natural log of the detected value and adding one prior to inferential analyses.

## Results

Participant baseline characteristics are shown in Table 1. Our participants averaged 56.2 years of age, and one-third self-reported Hispanic ethnicity (32%), with race self-reported as predominately white (80%). Our participants were highly educated with 60% having obtained at least a bachelor’s degree. Approximately half of our participants (49%) were fully employed and 36% reported as either retired or home-maker. The 94 participants had a mean time from breast cancer diagnosis to study entry of 5.9 years (SD = 5.0; range 2 months-22 years). Most participants had either invasive (45.9%) cancer or ductal carcinoma *in situ* (DCIS, 49.2%). Over 60% were diagnosed at Stage I (30.2%) or II (31.7%); 64.8% did not know their breast cancer subtype; and 64.4% did not know their breast cancer susceptibility gene mutation (BRCA1 or BRCA2) carrier status. Similarly, 64.1% were >2 years post-treatment. Therapy regimens included radiotherapy (78%) and chemotherapy (84%); all received surgical treatment, 65% reported receiving hormonal therapy, and 23% received Herceptin Antibody therapy (data not shown). Participants presented at baseline with moderate co-morbidities (2.3), were overweight (BMI = 28.8 kg/m^2^) and had very low cardiorespiratory capacity (19.8 ml O2/kg/min), less than the 10^th^ percentile for age and gender as reported by ACSM fitness categories (American College of Sports Medicine 2013).

**Table 1:** **Participant characteristics at baseline, mean standard deviation or n (%)**

**Baseline characteristic**	**All (N = 94)**	**YE (n = 31)**	**CE (n = 31)**	**C (n = 32)**	***p***
Age	56.2 (7.9)	56.7 (9.6)	57.6 (6.6)	54.4 (7.0)	.266
Co-Morbidity	2.3 (1.7)	2.1 (1.7)	2.1 (1.5)	2.7 (1.8)	.323
BMI	28.8 (6.7)	29.1 (6.7)	29.1 (6.2)	28.1 (7.3)	.810
VO2max	19.8 (5.1)	20.2 (5.6)	19.2 (4.9)	19.9 (5.0)	.737
Lymphedema					
Yes	19 (20%)	5 (16%)	6 (19%)	8 (25%)	
No	72 (77%)	25 (81%)	25 (81%)	22 (69%)	
Missing	3 (37%)	1 (3%)	1 (3%)	2 (6%)	
Ethnicity					
Hispanic	30 (32%)	10 (32%)	7 (23%)	13 (41%)	
Non-Hispanic	63 (67%)	21 (68%)	24 (77%)	18 (56%)	
Missing	1 (1%)			1 (1%)	
Race					
White	75 (79%)	26 (84%)	25 (81%)	24 (75%)	
African American	5 (5%)	--	2 (6%)	3 (9%)	
Asian	1 (5%)	1 (5%)	--	--	
Other	11 (11%)	4 (13%)	3 (10%)	4 (12%)	
Missing	2 (2%)	--	1 (3%)	1 (3%)	
Education					
High school diploma	8 (8%)	3 (10%)	2 (6%)	3 (9%)	
Technical	3 (3%)	3 (10%)	--	--	
Some college	23 (25%)	7 (23%)	7 (23%)	9 (28%)	
Bachelor’s degree	26 (28%)	10 (32%)	9 (29%)	7 (22%)	
Master’s degree	28 (30%)	6 (19%)	10 (32%)	12 (38%)	
Terminal degree (e.g.MD, PhD)	4 (4%)	2 (6%)	2 (6%)	--	
Missing	2 (2%)	--	1 (3%)	1 (3%)	
Employment Status					
Employed full time	46 (49%)	14 (45%)	15 (48%)	17 (53%)	
Employed part time	8 (8%)	1 (3%)	5 (16%)	2 (6%)	
Not working but seeking	2 (2%)	2 (6%)	--	--	
Not working not seeking	1 (1%)	--	--	1 (3%)	
Retired	22 (23%)	8 (26%)	8 (26%)	6 (19%)	
Homemaker	13 (13%)	6 (19%)	3 (10%)	4 (13%)	
Volunteer	1 (1%)	--	--	1 (3%)	
Missing	1 (1%)	--	--	1 (3%)	

Total % may not add up to 100% due to rounding. YE = Yoga-based exercise group; CE = Comprehensive exercise group; C = Comparison group.

Descriptive results, tests for mean differences (“pre” and “post”) and effect sizes specific to the YE group are shown in Table 2. Though weight remained essentially the same (+0.23 kg), significant improvements were seen in body composition with a reduction of % body fat (−3.00%, *d* = −0.44, p = .001). There were no significant changes in inflammatory serum bio-markers and the direction of change was opposite of expected.

**Table 2:** **Descriptive statistics for yoga group inflammatory biomarkers,** ***n*** **= 20**

**Biological marker**	**Pre range**	**Pre mean (SD)**	**Post range**	**Post mean (SD)**	**Expected direction**	P**	**Change score**	(95%CI)* change	**ES** ***
Weight (kg)	54.6-102.1	74.6 (14.8)	55.8-97.8	74.8 (14.8)	Decrease	.766	0.23	(−1.34, 1.79)	.02
BMI (kg/m^2^)	19.9-40.5	28.8 (6.5)	20.1-39.9	28.8 (6.0)	Decrease	.942	0.03	(−0.79, 0.85)	.00
Body Adiposity (%)	18.1-43.4	34.0 (6.8)	18.1-41.2	31.0 (6.5)	Decrease	**<.001**	−3.00	(−4.50, −1.50)	-.44
IL-6 (pg/mL)	1295.9- 10170.1	5442.8 (2799.8)	1388.1- 11668.6	5478.3 (3153.9)	Decrease	.836	35.49	(−1135.0, 1206.0)	.013
IL-8 (pg/mL)	274.0 - 2066.1	1309.5 (479.1)	59.5 - 2972.5	1483.3 (717.4)	Decrease	.930	141.77	(− 137.1, 420.0)	.294
TNFα (pg/mL)	1042.0- 7296.6	2206.9 (1472.7)	709.2 - 6227.0	2354.5 (1241.7)	Decrease	.227	184.61	(− 137.1, 420.0)	.129
CRP (μg/mL)	0.89 - 31.13	5.28 (6.95)	1.04 - 16.27	5.30 (4.35)	Decrease	.624	0.018	(− 3.65, 3.62)	.003

*95% confidence interval of the mean change score; **t test on transformed values for serum markers; ***ES = effect size = (“post” mean - “pre” mean)/standard deviation of “pre” score.

Data in boldface reflect significant differences at p<0.05.

As can be seen in Table 3, when comparing all groups, all participants lost body fat; however, the YE group lost the most with an average of 3.00%, with the CE group and C group losing 2.46% and 1.97%, respectively. Interestingly, though while losing the most % body fat, the YE group gained mass (0.23 kg), indicative of favorable changes in body composition when compared to the other groups.

**Table 3:** **Differences between groups change score (post minus pre) ANOVA, ANCOVAs**

	**All (n = 72) mean (SD)**	**YE (n = 20) mean (SD)**	**CE (n = 26) mean (SD)**	**C (n = 26) mean (SD)**	**Expected direction**	p*	p**	p***	**Post-hoc**
Weight (kg)	−0.09 (2.83)	0.23 (3.35)	−0.65 (2.91)	0.20 (2.29)	Decrease	.479	-	-	
BMI (kg/m^2^)	−0.02 (1.30)	0.03 (1.75)	−0.14 (1.27)	0.06 (0.91)	Decrease	.838	-	-	
Body Adiposity (%)	−2.43 (3.11)	−3.00 (3.21)	−2.46 (3.05)	−1.97 (3.14)	Decrease	.540	-	-	
IL-6 (pg/mL)	316.85 (2266.69)	35.49 (2353.78)	317.62 (3373,67)	557.28 (2558.23)	Decrease	.646	.606	.828	
IL-8 (pg/mL)	0.69 (514.23)	141.77 (579.25)	−52.10 (454.40)	−63.55 (516.38)	Decrease	953	.903	.943	
TNFα (pg/mL)	289.0 (1204.51)	184.61 (750.28)	860.74 (1620.38)	−212.13 (756.10)	Decrease	.057	.**036**	**.046**	**CE > C**
CRP (μg/mL)	−0.06 (11.2)	0.018 (7.77)	−0.37 (12.90)	0.20 (12.11)	Decrease	.490	440	.422	

YE = Yoga-based exercise group; CE = Comprehensive exercise group; C = Comparison group; *ANOVA on transformed values for serum markers; **ANCOVA controlling for BMI and VO2max; ***ANCOVA controlling for BMI, VO2max and baseline level of transformed value for serum marker. Data in boldface reflect significant differences at p<0.05.

There were no significant differences between groups in changes in inflammatory serum bio-markers. However, the CE group had changes in the expected direction for IL-8 and CRP and the C group had changes in the expected direction for IL-8 and TNFα. The only significant difference between groups was for TNFα where the CE differed from the C group. This was due to the unexpected increase in TNFα in the CE group and expected though not significant decrease in the C group.

## Discussion

Evidence continues to accumulate demonstrating the benefits of exercise on reducing morbidity and mortality while improving individual QOL and overall health (Schmitz et al. 2010; United States Department of Health and Human Services 2008; Haskell et al. 2007; Lichtenstein et al. 2006; United States Department of Health and Human Services 1996; Blair et al. 1989). These benefits apply to cancer survivors as well (Schmitz et al. 2010; Doyle et al. 2006; Speck et al. 2010; Holmes et al. 2005; Beasley et al. 2012). Adopting and maintaining a physically active lifestyle improves cancer survivors’ well-being (Courneya 2003), and reduces their risk of cardiovascular disease (LaCroix et al. 1996), noninsulin-dependent diabetes mellitus (Helmrich et al. 1991), osteoporosis (Devogelaer and de Deuxchaisnes 1993), and recurrent cancers (Giovannucci et al. 1995; Friedenreich and Rohan 1995). However, cancer survivors tend to decrease their level of physical activity after diagnosis and most never regain their former levels after treatment (Irwin et al. 2003; Courneya 1997; Irwin et al. 2004).

Thus, the need for exercise activities that engage breast cancer survivors is urgent. Moreover, the design of exercise activities specifically modified for the often compromised physical functioning of post-treatment breast cancer survivors is critical. The specificity of the design of a program increases its potential to optimize desired outcomes. In addition to aerobic and/or resistance exercise programs, yoga-based programs are starting to be used in this population. Here we reported on the inflammatory markers results for 20 post-treatment breast cancer survivors who safely completed a six-month structured Hatha yoga**-**based exercise trial and compared their results to groups randomized to ‘conventional’ comprehensive exercise (aerobic, resistance, flexibility) or a comparison group who chose their form(s) of exercise.

Our unexpected opposite effect, or no effect, of exercise on inflammatory marker levels has been previously observed by other groups, particularly for IL-6 (Payne et al. 2008; Jones et al. 2013; Rogers et al. 2013), and evidence is still generally considered preliminary (Ballard-Barbash et al. 2012). We plan to conduct future exercise interventions that examine inflammation-related serum markers under more closely-supervised and structured exercise conditions.

Participation in the trial was also associated with improvements in body composition (body fat loss of 3%) for the YE group, which had the most favorable change in body composition among the three groups. This is consistent with Littman and colleagues’ six month study where yoga participants had a significant favorable change in waist circumference compared to a wait list control. Similar to our results, those participants also did not lose weight (Littman et al. 2012). The change in body composition for our participants is important to note as obesity and obesity-associated endocrine output, including pro-inflammatory cytokines, has been associated with breast cancer recurrence risk (Chen et al. 2010; Demark-Wahnefried et al. 2012; Gilbert and Slingerland 2013; Morimoto et al. 2002; Simpson et al. 2013). Some studies suggest that the association of the physical activities’ effect on biological markers with breast cancer risk may not only be the direct effect of activity but also the result of a favorable effect on managing/reducing obesity (Irwin et al. 2011; McTiernan 2008; Ballard-Barbash et al. 2012; Irwin et al. 2005; McTiernan et al. 2003a; McTiernan et al. 2003b; McTiernan et al. 2006). Since the weight stayed virtually the same in our YE participants (+0.23 kg), the loss in body fat probably translated to a gain in lean mass, though in the absence of specific assessment techniques (e.g. dual-energy x-ray absorptiometry) we cannot be certain. A recent review described exercise effects on skeletal muscle-derived myokines, including IL-6, which increase release of other anti-inflammatory cytokines (Goh et al. 2014). This may help explain the IL-6 increase seen in our participants coupled with the hypothesized increase in lean muscle mass. Future studies focusing on skeletal muscle effects of exercise should elucidate this potential relationship.

This study has certain limitations and strengths that should be noted. First, interpretation of our results should be guarded because of the relatively small sample size (n = 20) of YE participants that completed our trial. As noted in Figure 1 we had 11 participants drop out of our study for various reasons - a relatively high attrition rate, equal to the other two groups combined. A contributing factor could be the requirement to attend classes at a specific yoga studio during specific times. Though we offered several times and dates to accommodate varying schedules**,** this constraint may have become a significant barrier to participation. Secondly, outside of those participants that attended the yoga studio classes we have no exact knowledge of the quality of yoga, if any, that our participants practiced outside of class. As mentioned, we did give participants a set of instructions and an audio CD with which to practice the specific protocol outside of the studio. Participants may or may not have done the yoga routine as instructed; moreover, any of the participants could have participated in other activities without our knowledge. Future efforts will include improving access to the yoga-based exercise**.**

Another limiting factor was that we did not assess dietary habits at either assessment. Moreover, we did not require participants to fast or refrain from exercise prior to blood draw. IL-6 levels, for example, can be transiently elevated for up to 24 hours after an acute bout of exercise (Rogers et al. 2013). These factors may have contributed to the unexpected results.

Nonetheless, our study has strengths as well. We randomized 94 breast cancer survivors into 3 different types of exercise programs, including yoga-based exercise, with relatively long duration (26 weeks). We are one of the few studies that had a stringent yoga-based exercise program designed specifically for post-treatment breast cancer survivors, which also assessed changes in inflammatory biological markers. Larger-scale studies that incorporate yoga-based exercise are warranted to determine optimal exercise protocols for specific cancer survivor populations.
